# Effect of surgical timing in outcomes in Hispanic patients after arthroscopic capsular release in diabetic and idiopathic adhesive capsulitis

**DOI:** 10.1016/j.jseint.2023.06.007

**Published:** 2023-07-03

**Authors:** Lucas De Virgilio-Salgado, David Deliz-Jimenez, Henry Ruberte, Francis Cedeño-Rodriguez, Gustavo Rivera-Rodriguez, Norman Ramírez, Antonio Soler-Salas, Efrain Deliz-Asmar

**Affiliations:** a Department of Orthopaedic Surgery, University of Puerto Rico, Medical Sciences Campus, San Juan, PR, USA; b General Surgery Department, University of Puerto Rico Medical Sciences Campus, San Juan, PR, USA; c University of Puerto Rico School of Medicine, University of Puerto Rico Medical Sciences Campus, San Juan, PR, USA; d Universidad Central del Caribe School of Medicine, Bayamon, PR, USA; e Orthopaedic Surgery Department, Mayagüez Medical Center, Mayagüez, PR, USA; f Department of Orthopaedic Surgery, Hospital HIMA San Pablo, Bayamon, PR, USA

**Keywords:** Adhesive capsulitis, Arthroscopic capsular release, Frozen shoulder, Timing, Diabetic adhesive capsulitis, Idiopathic adhesive capsulitis

## Abstract

**Background:**

Adhesive capsulitis of the shoulder is a painful and debilitating condition. While the majority of patients improve with conservative treatment, those who do not improve require surgery such as arthroscopic capsular release (ACR) for symptom relief. However, there is limited literature regarding the optimal timeframe to proceed with surgery.

**Methods:**

This retrospective cohort evaluated 134 Hispanic patients who underwent ACR for the treatment of adhesive capsulitis. Patients were divided into an early and a delayed treatment group that included all patients. Patients were then divided into diabetic and idiopathic subgroups. Early vs. delayed treatment outcomes (forward flexion, external rotation, Visual Analog Scale pain scores, and recurrence requiring reoperation) were assessed in all patients and in each subgroup.

**Results:**

No statistically significant differences were found between the early and delayed release groups in postoperative forward flexion, external rotation, pain intensity scores, and recurrence requiring reoperation at 1 month, 3 months, and 6 months of follow-up in the all-patient group. In the idiopathic frozen shoulder subgroup, no significant differences were observed in postoperative forward flexion, external rotation, pain intensity scores, and recurrence requiring reoperation at 1 month, 3 months, and 6 months of follow-up. In the diabetic frozen shoulder subgroup, no significant differences were observed in postoperative forward flexion, external rotation, pain intensity scores, and recurrence requiring reoperation at 1 month and 6 months of follow-up visits.

**Conclusions:**

There was no difference in outcomes following ACR for adhesive capsulitis between patients who underwent early release vs. delayed release. There were no significant differences in outcomes between early and delayed arthroscopic release in patients with a history of diabetes mellitus.

Adhesive capsulitis of the shoulder, also known as frozen shoulder or arthrofibrosis, is a painful and debilitating condition. Primary (idiopathic) adhesive capsulitis occurs spontaneously with no associated trauma to the shoulder, affecting 3%-5% of the general population.^17^ Secondary frozen shoulder is associated with other etiologies such as metabolic disorders (diabetes mellitus [DM], hypothyroidism), post-trauma, postsurgical, cervical radiculopathy, and myocardial infarction.^13^^,^^18^ Among all these factors, DM has been described as the most predominant risk factor for secondary adhesive capsulitis in both genders.^20^ The incidence of diabetic frozen shoulder is up to 30% and has been reported to be more aggressive and has a higher resistance to treatment than idiopathic adhesive capsulitis.^5^^,^^32^ Additionally, diabetic patients do not always follow the natural history of idiopathic adhesive capsulitis and often require some intervention.^29^

Adhesive capsulitis is mostly a self-limiting condition. The natural history of idiopathic frozen shoulder has been divided into three stages, each lasting about 6 months.^28^ It begins with the freezing phase characterized by an abrupt and severe onset of shoulder pain. The frozen phase starts next, where pain improves, but the range of motion becomes more limited. Finally, the thawing phase, in which a gradual and slow improvement in range of motion is observed, but this phase can take between 12 and 42 months.^28^

Most patients with adhesive capsulitis are successfully managed with conservative treatment, which includes a course of physical therapy and range of motion exercises with or without glenohumeral corticosteroid injections, hydrodylation, and oral anti-inflammatory medications.^23^ If conservative treatment fails, manipulation under anesthesia and arthroscopic or open capsular release can be considered.^13^^,^^23^ Arthroscopic capsular release (ACR) has been shown to improve functional outcomes and reduce pain severity in patients with idiopathic and diabetic frozen shoulder in the short and long term.^1^^,^^2^^,^^9^^,^^14^^,^^19^ However, worse outcomes have been shown in diabetic patients compared to idiopathic frozen shoulder patients after surgical intervention.^19^

Although extensive research has been done regarding idiopathic and diabetic frozen shoulder management, there is no universal treatment algorithm or consensus regarding the optimal timeframe to proceed with surgery in either group. Few studies have assessed the relationship between the timing of ACR and functional outcomes. It remains to be determined which of the three phases of frozen shoulder an ACR would provide the best functional outcomes. Previous studies have suggested delayed surgical treatment as a better option for patients with idiopathic frozen shoulder.^7^^,^^8^^,^^22^^,^^26^ The popular belief is that surgery in the early stages of the disease may result in poorer outcomes as it is associated with more severe synovitis.^10^ In 2019, Rizvi contradicted previous studies and reported improved functional outcomes in patients who undergo early ACR compared to delayed capsular release in patients with idiopathic frozen shoulder.^27^ However, studies have not focused on optimal timeframes for ACR in patients with diabetic adhesive capsulitis, which is considered a more severe disease often requiring earlier surgical treatment.^29^^,^^31^

The objective of our study was to compare outcomes (Visual Analog Scale [VAS] pain score and range of motion) of early vs. delayed ACR in Hispanic patients with frozen shoulder and to analyze further the outcomes of the timing of the release in patients with idiopathic and diabetic adhesive capsulitis. Since this has not been evaluated in Hispanic patients with adhesive capsulitis, and Hispanics are more likely to have adhesive capsulitis and less likely to have surgery for adhesive capsulitis, it is pertinent to focus on this patient population.^11^ Our hypothesis was that (1) ACR performed in Hispanic patients during the early phase of frozen shoulder would provide poorer outcomes when compared to delayed release and (2) early release would have worse improvements in outcomes compared to delayed release when subanalyzing the diabetic and idiopathic frozen shoulder cohort.

## Methods

This retrospective cohort study evaluated 134 Hispanic patients surgically treated for a diagnosis of adhesive capsulitis from January 2013 to January 2020 by a single fellowship-trained shoulder and elbow surgeon with more than 25 years of experience. A subject list was generated using specific procedure codes (Current Procedural Terminology code 29825) for ACR to treat adhesive capsulitis diagnosis.

Inclusion criteria included patients aged more than 21 years, patients with a clinical diagnosis of idiopathic or diabetic adhesive capsulitis, patients who received an ACR, and patients with a minimum of 6 months of follow-up after surgery. Exclusion criteria included patients aged less than 21 years, the presence of significant glenohumeral arthritis observed radiologically, the presence of rotator cuff tear, the presence of reactive arthritis, calcific tendinitis, previous shoulder surgery, previous infection of the shoulder joint, history of significant trauma, fracture or dislocation of the affected shoulder, and patients with bilateral shoulder involvement. Patients with no reported range of motion or VAS score values for at least 6 months were not included in the study (61 patients with less than 6 months of follow-up were excluded).

### Diagnosis and indications for arthroscopic capsular release

Patients were diagnosed with adhesive capsulitis if they met the following criteria: range of motion limited in all planes with a direct comparison to the contralateral shoulder, radiographic findings that do not show osteoarthritic changes, and patients with an magnetic resonance imaging (MRI) that ruled out major cuff pathologies (all patients had MRI performed). MRI may also show a thickened capsule, but this was not required for a diagnosis to be made. Patients underwent ACR if they met the following criteria: Pain with motion and failure of conservative treatment (physiotherapy, daily home exercise program, pain management, periarticular injections) or severe pain causing disability.

### Surgical technique

Patients received interscalene block and were placed in the beach chair position. Bony landmarks were identified and marked after standard draping. The shoulder joint was distended with 60 mL of Normal Saline Solution using a spinal needle (18 g) inserted posteriorly. The posterior portal was then established, and the joint was inspected sequentially. The anterior portal was then established with a cannula. The anterior capsular release was performed with a Vaporizer and débridement using the full radius resector. The rotator interval was released with a vaporizer and a full radius resector. Portals were interchanged using the Wissinger rod. The posterior capsular release was performed with Vaporizer. Releases were performed from 12 o’clock to 5 o’clock and from 5 o’clock to 12 o’clock. The medial glenohumeral ligament is liberated in this release. At the end of the procedure, gentle manipulation was done to release the inferior capsule by gently elevating the arm combined with external and internal rotation until soft tissue gives way. Progressive gains in range of motion were observed. The shoulder joint was distended several times with normal saline and epinephrine 1% solution to help control any residual bleeding, with a total of 150-200 mL of solution used in sequential opening and closing of cannula valves at this end stage of the procedure. The scope was then removed and redirected to the subacromial space posteriorly for inspection to rule out further pathology. Bursectomy is only performed if there was evidence of impingement or marked bursal reaction. Lysis of subacromial or subdeltoid adhesions was performed if present. The portals were closed with Prolene 3-0, sterile dressings were applied, and the arm was placed in a simple sling.

### Postoperative protocol

Patients were oriented in the recovery room to begin passive and active range of motion from day 1 postsurgery. All patients were evaluated in the clinics at 1 month, 3 months, and 6 months after surgery. Physical therapy was coordinated for at least 6 weeks. The patients also received further instructions regarding home stretching exercises.

### Outcome measures

Patients were divided into two groups based on the duration of their symptoms before ACR was performed. Patients were divided into an early (<10 months of symptom duration) and a delayed treatment group (≥10 months of symptom duration). Cutoffs for groups were based on adhesive capsulitis disease progression and the cutoff point used in prior studies.^27^^,^^29^ Patients were then divided into diabetic and idiopathic subgroups. Diabetic status was obtained from the patient’s chart. Early vs. delayed treatment outcomes were assessed in each subgroup.

Primary outcome measures were (1) changes forward flexion (FF) and external rotation (ER) after ACR, (2) change in VAS score, and (3) recurrence requiring reoperation (persistent limitation 6 months postrelease and surgeon judgment following patient complaints). Preoperative range of motion was measured with a goniometer by an experienced assistant and a preoperative VAS score was obtained during the encounter before ACR. The patient was evaluated at clinics at 1 month, 3 months, and 6 months after surgical intervention. Range of motion (FF and ER) was measured with a goniometer by the surgeon’s assistant. Pain levels were assessed at each clinical visit with VAS scores, where patients were asked by the surgeon’s assistant to rate their pain on a scale of 0 (absence of pain) to 10 (worst pain of their life).

Study groups and variables were compared to determine if statistical differences were present. The Student’s *t*-test was used to compare continuous variables, while the Chi-square test was employed to measure differences in categorical values. A *P* value of < .05 was considered statistically significant. Microsoft Excel (Microsoft Corp., Redmond, WA, USA) and SPSS (IBM Corp., Armonk, NY, USA) software were used for statistical analyses. The Institutional Review Board from the University of Puerto Rico Medical Science Campus approved this study.

## Results

### Overall patient demographics

A total of 134 Hispanic patients who underwent ACR for adhesive capsulitis met the inclusion criteria. The mean age of the study population was 57.4 years (standard deviation 8.88), with 74.6% (100/134) of patients being female. The mean population Body Mass Index was 27.83 (standard deviation 4.80). Among all patients, 43% (58/134) had a diagnosis of DM.

### Early release vs. delayed release patient demographics

Our patient cohort's median length of symptoms before surgery was 10 months (interquartile range 6.25). The median length of symptoms before surgery for the early release group was 6 months (range 2-9 months), and the median length of symptoms before surgery in the delayed treatment group was 13 months (range 10-62 months). Surgical timing was divided into the following ranges: 0-3 months: 4 patients; 4-6 months: 29 patients; 7-9 months: 36 patients; and >10 months: 65 patients. The early treatment group comprised 69 patients with a mean age of 58.35, with 73.9% (51/69) females. The delayed treatment group included 65 patients with a mean age of 56.44, with 75.4% female. There was no statistically significant difference between the groups regarding age, sex, and DM. The data can be seen in Table I.

**Table I:** Group demographics.

Factor	Operative treatment <10 mo (N = 69)	Operative treatment >10 mo (N = 65)	*P* value
Age	58.35	56.44	.108
Sex			.845
Male	18	16	
Female	51	49	
Diabetes Mellitus			.692
Yes	31	27	
No	38	38	
BMI	27.51	28.13	.226

*BMI*, body mass index.

### Early release vs. late release outcomes in all patients

Preoperative range of motion was compared between groups. There was no statistically significant difference between early and late release groups regarding preoperative FF (*P* value = .183) and ER (*P* value = .209). Postoperatively, no statistically significant differences were recorded between groups with flexion at 1-month follow-up (*P* value = .300), flexion at 3-month follow-up (*P* value = .103), flexion at 6-month follow-up (*P* value = .257), external rotation at 1-month follow-up (*P* value = .244), external rotation at 3-month follow-up (*P* value = .411), external rotation at 6-month follow-up (*P* value = .496), and in recurrence requiring surgical management (*P* value = .754).

Preoperative pain scores were compared between both groups, and no significant difference was found (*P* value = .120). Postoperatively, there were no statistically significant differences in VAS pain scores at 1-month, 3-month, or 6-month follow-up periods (*P* value = .098, *P* value = .276, and *P* value = .894, respectively). The data can be seen in Table II.

**Table II:** Outcome comparisons between early and delayed arthroscopic capsular release.

Outcomes	Operative treatment <10 mo (N = 69)	Operative treatment >10 mo (N = 65)	*P* value
Preoperative VAS Pain Score	8.89 ± 1.41	8.61 ± 1.32	.120
VAS Score 1 mo follow-up	4.19 ± 1.57	3.83 ± 1.62	.098
VAS Score 3 mo follow-up	3.26 ± 1.4	3.02 ± 1.18	.276
VAS Score 6 mo follow-up	2.25 ± 1.38	2.28 ± 1.26	.894
Preoperative Flexion (°)	99.64 ± 22.00	102.77 ± 17.55	.183
Flexion 1 mo follow-up (°)	119.35 ± 15.09	120.92 ± 19.42	.300
Flexion 3 mo follow-up (°)	128.99 ± 15.11	132.38 ± 15.91	.103
Flexion 6 mo follow-up (°)	140.26 ± 18.67	142.27 ± 16.65	.257
Preoperative External Rotation (°)	25.51 ± 16.21	23.38 ± 13.81	.209
External Rotation 1 mo follow-up (°)	34.64 ± 9.68	36.00 ± 12.82	.244
External Rotation 3 mo follow-up (°)	43.99 ± 13.86	44.54 ± 14.41	.411
External Rotation 6 mo follow-up (°)	54.13 ± 14.99	54.15 ± 15.19	.496
Recurrence requiring surgical management			.754
Yes	12	10	
No	57	55	

*VAS*, visual analog scale.

### Early vs. delayed release in idiopathic frozen shoulder

**Figure 1:**
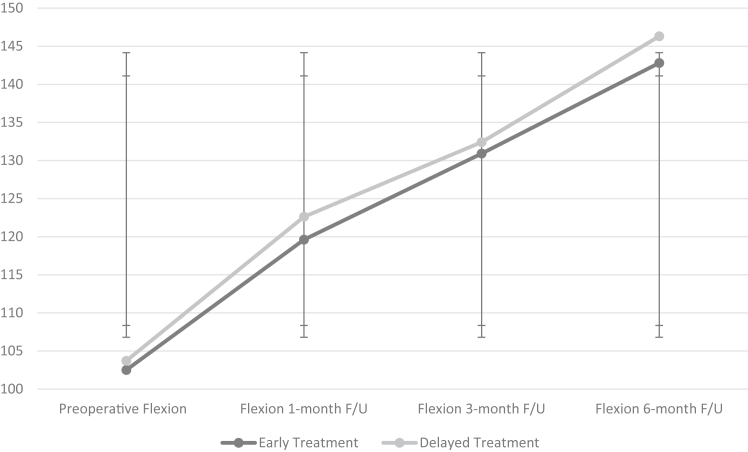
Flexion in early vs. delayed arthroscopic capsular release (ACR) in idiopathic adhesive capsulitis. Flexion observed preoperatively and postoperatively at 1 month, 3 months, and 6 months of follow-up are tracked for each group. The early treatment group consists of patients who underwent ACR before 10 months from symptom onset, and the delayed treatment group consists of patients who underwent ACR at or after 10 months from symptom onset. The x-axis represents the time at which flexion was measured, and the y-axis represents degrees of movement.

**Figure 2:**
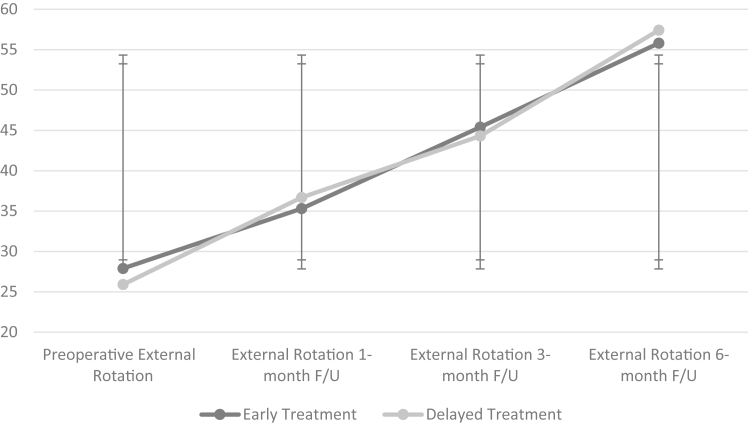
External rotation in early vs. delayed arthroscopic capsular release (ACR) in idiopathic adhesive capsulitis. External rotation observed preoperatively and postoperatively at 1 month, 3 months, and 6 months of follow-up are tracked for each group. The early treatment group consists of patients who underwent ACR before 10 months from symptom onset, and the delayed treatment group consists of patients who underwent ACR at or after 10 months from symptom onset. The x-axis represents the time at which external rotation was measured, and the y-axis represents degrees of movement.

The idiopathic frozen shoulder subgroup in the study population consisted of 76 patients with a mean age of 56.5 years and 87% were female. In this subgroup, 38 underwent early capsular release and 38 underwent delayed release. Preoperative FF and ER also showed no statistically significant differences (*P* value = .405 and *P* value = .272, respectively). Postoperatively, no statistically significant differences were recorded between groups with flexion at 1-month follow-up (*P* value = .242), flexion at 3-month follow-up (*P* value = .342), and flexion at 6-month follow-up (*P* value = .183). No significant differences were observed in postoperative external rotation at 1-month follow-up (*P* value = .290), external rotation at 3-month follow-up (*P* value = .365), external rotation at 6-month follow-up (*P* value = .325), and in recurrence requiring surgical management (*P* value = .723) (Figs. 1 and 2).

Preoperative VAS pain score in the early vs. delayed groups showed no statistically significant difference (*P* value = .160). Postoperatively, there were no statistically significant differences in VAS pain scores at the 1-month, 3-month, or 6-month follow-up periods (*P* value = .164, *P* value = .400, and *P* value = .076, respectively). The data can be seen in Table III.

**Table III:** Early vs. delayed arthroscopic release outcomes in nondiabetic patients.

Outcomes	Operative treatment <10 mo (N = 38)	Operative treatment >10 mo (N = 38)	*P* value
Preoperative VAS Pain Score	8.7 ± 1.6	8.3 ± 1.3	.160
VAS Score 1 mo follow-up	4.0 ± 1.5	3.7 ± 1.5	.164
VAS Score 3 mo follow-up	3.0 ± 1.5	3.1 ± 1.2	.400
VAS Score 6 mo follow-up	1.9 ± 1.4	2.4 ± 1.3	.076
Preoperative Flexion (°)	102.5 ± 23.3	103.7 ± 19.2	.405
Flexion 1 mo follow-up (°)	119.6 ± 13.9	122.6 ± 22.5	.242
Flexion 3 mo follow-up (°)	130.9 ± 15.6	132.4 ± 15.3	.342
Flexion 6 mo follow-up (°)	142.8 ± 19.8	146.3 ± 12.7	.183
Preoperative External Rotation (°)	27.9 ± 14.3	25.9 ± 13.8	.272
External Rotation 1 mo follow-up (°)	35.3 ± 10.7	36.7 ± 11.9	.290
External Rotation 3 mo follow-up (°)	45.4 ± 13.7	44.3 ± 12.7	.365
External Rotation 6 mo follow-up (°)	55.8 ± 14.9	57.4 ± 15.4	.325
Recurrence requiring surgical management			.723
Yes	5	4	
No	33	34	

*VAS*, visual analog scale.

### Early vs. delayed release in diabetic frozen shoulder

**Figure 3:**
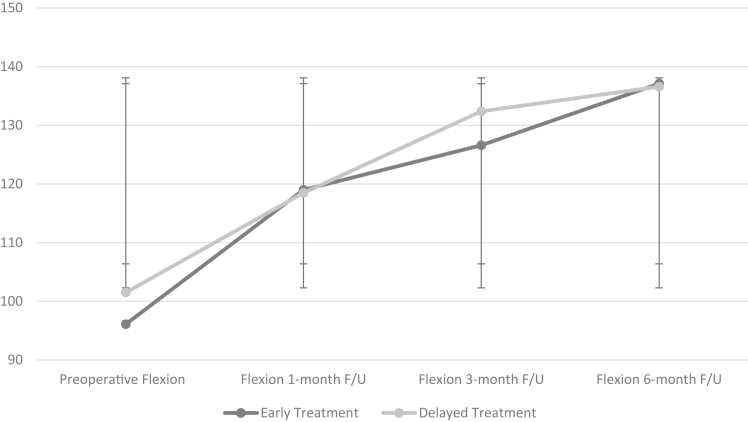
Flexion in early vs. delayed arthroscopic capsular release (ACR) in diabetic adhesive capsulitis. Flexion observed preoperatively and postoperatively at 1 month, 3 months, and 6 months of follow-up are tracked for each group. The early treatment group consists of patients who underwent ACR before 10 months from symptom onset, and the delayed treatment group consists of patients who underwent ACR at or after 10 months from symptom onset. The x-axis represents the time at which flexion was measured, and the y-axis represents degrees of movement.

**Figure 4:**
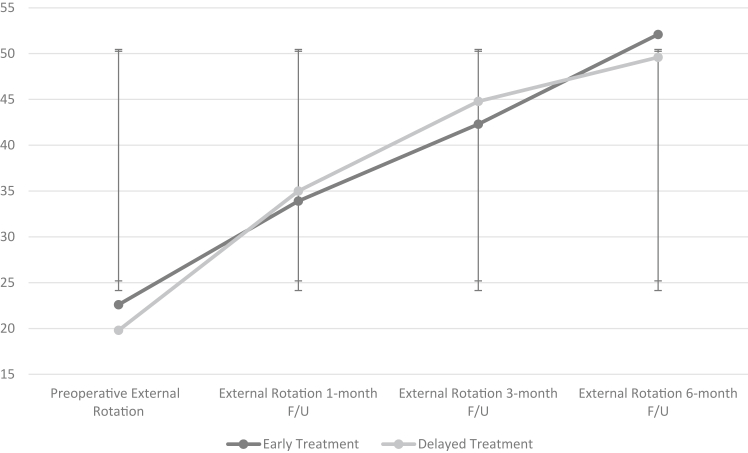
External rotation in early vs. delayed arthroscopic capsular release (ACR) in diabetic adhesive capsulitis. External rotation observed preoperatively and postoperatively at 1 month, 3 months, and 6 months of follow-up are tracked for each group. The early treatment group consists of patients who underwent ACR before 10 months from symptom onset, and the delayed treatment group consists of patients who underwent ACR at or after 10 months from symptom onset. The x-axis represents the time at which external rotation was measured, and the y-axis represents degrees of movement.

The diabetic subgroup in the study population consisted of 58 patients with a mean age of 58.6 years, and 59% were female. In this subgroup, 31 underwent early capsular release and 27 underwent delayed release. Preoperative FF and ER showed no statistically significant differences (*P* value = .131 and *P* value = .257, respectively). Postoperatively, there were no statistically significant differences in FF at 1-month, 3-month, and 6-month follow-up visits between groups (*P* value = .450, *P* value = .083, and *P* value = .456, respectively). There were no statistically significant differences in external rotation at 1-month, 3-month, and 6-month follow-up visits postoperatively between groups (*P* value = .354, *P* value = .265, and *P*-value = .262, respectively). Patients with a history of diabetes did not have statistically significant differences in recurrence requiring reoperation between the early and delayed treatment groups (*P* value = .974) (Figs. 3 and 4).

Preoperative VAS pain scores in the early vs. delayed cohorts showed no statistically significant difference (*P* value = .300). Postoperatively, there were no statistically significant differences in VAS scores between both groups at 1 month and at the 6-month follow-up visit (*P* value = .218 and *P* value = .065, respectively). The data can be seen in Table IV.

**Table IV:** Early vs. delayed arthroscopic release outcomes in diabetic patients.

Outcomes	Operative treatment <10 mo (N = 31)	Operative treatment >10 mo (N = 27)	*P* value
Preoperative VAS Pain Score	9.1 ± 1.1	8.9 ± 1.2	.300
VAS Score 1 mo follow-up	4.4 ± 1.6	4.0 ± 1.8	.218
VAS Score 3 mo follow-up	3.6 ± 1.3	2.9 ± 1.1	**.021**
VAS Score 6 mo follow-up	2.6 ± 1.3	2.1 ± 1.2	.065
Preoperative Flexion (°)	96.13 ± 20.1	101.5 ± 15.1	.131
Flexion 1 mo follow-up (°)	119.0 ± 16.6	118.5 ± 13.9	.450
Flexion 3 mo follow-up (°)	126.6 ± 14.4	132.4 ± 17.0	.083
Flexion 6 mo follow-up (°)	137.1 ± 17.0	136.6 ± 19.8	.456
Preoperative External Rotation (°)	22.6 ± 18.1	19.8 ± 13.2	.257
External Rotation 1 mo follow-up (°)	33.9 ± 8.4	35.0 ± 14.1	.354
External Rotation 3 mo follow-up (°)	42.3 ± 14.1	44.8 ± 16.7	.265
External Rotation 6 mo follow-up (°)	52.1 ± 15.1	49.6 ± 14.0	.262
Recurrence requiring surgical management			.974
Yes	7	6	
No	24	21	

*VAS*, visual analog scale.

## Discussion

Hispanic patients in the early group had a median time from symptom onset to surgery of 6 months (range: 2-9 months), and the delayed treatment group had a median time from symptom onset to surgery of 13 months (range: 10-12 months). The early treatment cohort had similar improvements in FF, ER, pain intensity, and recurrence rates when compared to the delayed (≥10 months) treatment group. No significant differences were found between the two groups, suggesting that early treatment for adhesive capsulitis is as effective as delayed treatment in Hispanic patients with frozen shoulder. These results contradict the common belief that surgery in the early stages of the condition results in poorer outcomes due to the associated inflammatory process and severe synovitis. It also questions the need to delay surgery for 9-12 months after symptom onset, as recommended by previous authors.^26^ In the study by Levine in 2007, they describe that patients who undergo successful nonoperative management average around 4 months, while those who require surgery have undergone an average of 12 months of failed nonoperative management. However, our results suggest that patients who do not improve with nonoperative methods study should not delay operative management for 12 months and can safely opt for earlier ACR.^15^ Our earliest time between symptom onset and surgery was 8 weeks. The findings reinforce that an early surgical intervention, as suggested by previous studies, would be safe in Hispanic patients with frozen shoulder.^10^^,^^15^^,^^27^

While the optimal timing for ACR in patients with adhesive capsulitis has been heavily debated, recent studies suggest no difference in clinical outcomes between early and delayed surgical management. Our results aligned with these studies and demonstrated no significant differences in outcomes (flexion, ER, pain, recurrence requiring operation) between early and delayed release. Nevertheless, the decision remains patient-specific, as comorbidities, risk factors, and patient symptomatology must be considered. Since adhesive capsulitis that is diagnosed early may have a favorable course with conservative treatment, early surgical management for potentially self-resolving conditions introduces issues such as cost-effectiveness and surgical risks.^12^ The FROST study demonstrated comparable outcomes between manipulation under anesthesia, ACR, and physiotherapy.^25^ ACR was associated with higher risks, and manipulation under anesthesia was the most cost-effective. However, there were higher recurrence rates observed in non-ACR treatment modalities.^25^ Yet, early treatment may translate into less muscle atrophy, faster recovery and return to function, and better patient satisfaction given the more rapid symptom relief. These are important questions that will benefit from further research.

When comparing outcomes of ACR in idiopathic vs. diabetic frozen shoulder, both cohorts showed significant improvement in flexion, ER, and pain scores. These improvements are comparable to those reported by other studies.^1^^,^^10^^,^^19^^,^^27^ Also, no significant differences were found when comparing early and delayed release outcomes in both subgroups. Thus, while diabetic patients have been reported to have worse results after ACR when compared to idiopathic frozen shoulder patients, in our study, DM does not affect outcomes in early vs. delayed release.^19^^,^^21^

The prevalence of DM in Hispanics in the United States is higher than in other races.^3^^,^^24^ In this study, the prevalence of DM among patients undergoing ACR was 43%, almost double the percentage reported in a prior study (22%) that discussed the epidemiology of patients undergoing ACR for idiopathic adhesive capsulitis.^11^ These results, combined with the knowledge that Hispanics are less likely to undergo surgical treatment for adhesive capsulitis, highlight the importance of this study focused on a Hispanic population. Our results demonstrate that diabetic and nondiabetic Hispanic patients can safely opt for early surgical release, an important finding in a population with low surgical rates despite a higher prevalence of adhesive capsulitis. Although previous studies have reported diabetic patients with adhesive capsulitis to have worse flexion at 3 months and 6 months postsurgery and worse external rotation at 6 months postsurgery,^4^^,^^6^^,^^19^^,^^32^ our results suggest that surgical management does not need to be delayed in diabetic patients. Furthermore, literature has reported that while objective outcomes are inferior in diabetic patients, patient satisfaction between diabetic patients and nondiabetic patients regarding ACR is equal, with similar symptom relief reported.^16^ This is an exciting avenue to explore further since symptom relief is a significant reason; early surgical treatment is chosen instead of a more conservative, long-term approach. Furthermore, studies that investigate the severity of diabetes via hemoglobin A1C measurements and its association with adhesive capsulitis symptomatology and post-ACR outcomes must be explored to continue to understand the value of earlier surgical management.

This study has several limitations. The main limitation is the retrospective nature of the study. Data were obtained from a single surgeon and included only Hispanic patients. Hence, results may not be fully generalizable to a broader population. The cutoff date between early and delayed release was chosen due to previous studies which have used similar cutoff points in their analyses.^27^^,^^30^ Other cutoff dates indicating early or delayed treatment may be used, leading to different results. The absence of validated patient-reported outcome questionnaires is also a limitation and future studies including these would be of value. This study did not measure internal rotation, which would have provided more accurate results between groups. Additionally, we did not differentiate between active and passive range of motion, which would improve accuracy of results and measurements. Finally, patients were followed up for 6 months. Thus, other outcomes may be observed if patients receive follow-up for a more prolonged period.

## Conclusion

There was no difference in outcomes following ACR for adhesive capsulitis between Hispanic patients who underwent early release (<10 months) vs. delayed release (≥10 months). There were no significant differences in outcomes between early and delayed arthroscopic release in patients with a history of DM, suggesting diabetic patients do not need to wait to undergo arthroscopic release.

## Disclaimers

Funding: The authors declare that no funds, grants, or other support were received during the preparation of this manuscript.

Conflicts of interest: The authors, their immediate families, and any research foundation with which they are affiliated have not received any financial payments or other benefits from any commercial entity related to the subject of this article.

## References

[1] Barnes C.P., Lam P.H., Murrell G.A. (2016). Short-term outcomes after arthroscopic capsular release for adhesive capsulitis. J Shoulder Elbow Surg.

[2] Berghs B.M., Sole-Molins X., Bunker T.D. (2004). Arthroscopic release of adhesive capsulitis. J Shoulder Elbow Surg.

[3] Cheng Y.J., Kanaya A.M., Araneta M.R.G., Saydah S.H., Kahn H.S., Gregg E.W. (2019). Prevalence of diabetes by race and ethnicity in the United States, 2011-2016. JAMA.

[4] Cho C.H., Kim D.H., Lee Y.K. (2016). Serial comparison of clinical outcomes after arthroscopic capsular release for refractory frozen shoulder with and without diabetes. Arthroscopy.

[5] Dias R., Cutts S., Massoud S. (2005). Frozen shoulder. BMJ.

[6] Dyer B.P., Burton C., Rathod-Mistry T., Blagojevic-Bucknall M., van der Windt D.A. (2021). Diabetes as a prognostic factor in frozen shoulder: a systematic review. Arch Rehabil Res Clin Transl.

[7] Elhassan B., Ozbaydar M., Massimini D., Higgins L., Warner J.J. (2010). Arthroscopic capsular release for refractory shoulder stiffness: a critical analysis of effectiveness in specific etiologies. J Shoulder Elbow Surg.

[8] Fernandes M.R. (2013). Arthroscopic capsular release for refractory shoulder stiffness. Rev Assoc Med Bras (1992).

[9] Hand C., Clipsham K., Rees J.L., Carr A.J. (2008). Long-term outcome of frozen shoulder. J Shoulder Elbow Surg.

[10] Hasegawa A., Mihata T., Fukunishi K., Neo M. (2021). Does the timing of surgical intervention impact the clinical outcomes and overall duration of symptoms in frozen shoulder?. J Shoulder Elbow Surg.

[11] Kingston K., Curry E.J., Galvin J.W., Li X. (2018). Shoulder adhesive capsulitis: epidemiology and predictors of surgery. J Shoulder Elbow Surg.

[12] Kraal T., Hekman K., van den Bekerom M.P.J. (2020). What is the right timing for arthroscopic capsular release of a frozen shoulder? Letter to the Editor. Orthop J Sports Med.

[13] Le H.V., Lee S.J., Nazarian A., Rodriguez E.K. (2017). Adhesive capsulitis of the shoulder: review of pathophysiology and current clinical treatments. Shoulder Elbow.

[14] Le Lievre H.M., Murrell G.A. (2012). Long-term outcomes after arthroscopic capsular release for idiopathic adhesive capsulitis. J Bone Joint Surg Am.

[15] Levine W.N., Kashyap C.P., Bak S.F., Ahmad C.S., Blaine T.A., Bigliani L.U. (2007). Nonoperative management of idiopathic adhesive capsulitis. J Shoulder Elbow Surg.

[16] Lyhne J.M., Jacobsen J.R., Hansen S.J., Jensen C.M., Deutch S.R. (2019). Diabetic and non-diabetic patients report equal symptom relief after arthroscopic capsular release of frozen shoulder. J Clin Orthop Trauma.

[17] Manske R.C., Prohaska D. (2008). Diagnosis and management of adhesive capsulitis. Curr Rev Musculoskelet Med.

[18] McAlister I., Sems S.A. (2016). Arthrofibrosis after periarticular fracture Fixation. Orthop Clin North Am.

[19] Mehta S.S., Singh H.P., Pandey R. (2014). Comparative outcome of arthroscopic release for frozen shoulder in patients with and without diabetes. Bone Joint J.

[20] Milgrom C., Novack V., Weil Y., Jaber S., Radeva-Petrova D.R., Finestone A. (2008). Risk factors for idiopathic frozen shoulder. Isr Med Assoc J.

[21] Mubark I.M., Ragab A.H., Nagi A.A., Motawea B.A. (2015). Evaluation of the results of management of frozen shoulder using the arthroscopic capsular release. Ortop Traumatol Rehabil.

[22] Neviaser A.S., Hannafin J.A. (2010). Adhesive capsulitis: a review of current treatment. Am J Sports Med.

[23] Neviaser A.S., Neviaser R.J. (2011). Adhesive capsulitis of the shoulder. J Am Acad Orthop Surg.

[24] Pérez C.M., Soto-Salgado M., Suárez E., Guzmán M., Ortiz A.P. (2015). High prevalence of diabetes and prediabetes and their coexistence with cardiovascular risk factors in a Hispanic Community. J Immigr Minor Health.

[25] Rangan A., Brealey S.D., Keding A., Corbacho B., Northgraves M., Kottam L. (2020). Management of adults with primary frozen shoulder in secondary care (UK FROST): a multicentre, pragmatic, three-arm, superiority randomised clinical trial. Lancet.

[26] Redler L.H., Dennis E.R. (2019). Treatment of adhesive capsulitis of the shoulder. J Am Acad Orthop Surg.

[27] Rizvi S.M., Harisha A.J., Lam P.H., Murrell G.A.C. (2019). Factors affecting the outcomes of arthroscopic capsular release for idiopathic adhesive capsulitis. Orthop J Sports Med.

[28] Tasto J.P., Elias D.W. (2007). Adhesive capsulitis. Sports Med Arthrosc Rev.

[29] Whelton C., Peach C.A. (2018). Review of diabetic frozen shoulder. Eur J Orthop Surg Traumatol.

[30] Wong P.L., Tan H.C. (2010). A review on frozen shoulder. Singapore Med J.

[31] Yanlei G.L., Keong M.W., Tijauw Tjoen D.L. (2019). Do diabetic patients have different outcomes after arthroscopic capsular release for frozen shoulder?. J Orthop.

[32] Zreik N.H., Malik R.A., Charalambous C.P. (2016). Adhesive capsulitis of the shoulder and diabetes: a meta-analysis of prevalence. Muscles Ligaments Tendons J.

